# Experimental evidences of the NO action on a recombinant PrxII F from pea plant and its effect preventing the citrate synthase aggregation

**DOI:** 10.1016/j.dib.2015.02.009

**Published:** 2015-02-26

**Authors:** Daymi Camejo, Ana Ortiz-Espín, Juan J. Lázaro, María C. Romero-Puertas, Alfonso Lázaro-Payo, Francisca Sevilla, Ana Jiménez

**Affiliations:** a CEBAS-CSIC, Department of Stress Biology and Plant Pathology, E-30100 Murcia, Spain; b EEZ-CSIC, Department of Biochemistry, Cellular and Molecular Biology of Plants, E-18080 Granada, Spain

**Keywords:** Citrate synthase, Oligomerization, PrxII F, *S*-nitrosylation

## Abstract

*S*-nitrosylation is emerging as a key post-translational protein modification for the transduction of NO as a signaling molecule in plants. This data article supports the research article entitled “Functional and structural changes in plant mitochondrial PrxII F caused by NO” [1]. To identify the Cys residues of the recombinant PrxII F modified after the treatment with *S*-nitrosylating agents we performed the LC ESI–QTOF tandem MS and MALDI peptide mass fingerprinting analysis. Change in *A*_650 nm_ was monitored to estimate the thermal aggregation of citrate synthase in the presence *S*-nitrosylated PrxII F. The effect of the temperature on the oligomerization pattern and aggregation of PrxII F was analysed by SDS-PAGE and changes in absorbance at 650 nm, respectively.

## Specifications table

**Table:** 

Subject area	Biology
More specific subject area	*Biochemistry, proteomic*
Type of data	*Graph, figure*
How data was acquired	*Mass spectrometry (Eksigent 1D-nanoHPLC) coupled to a 5600 Triple TOF QTOF mass spectrometer (ABSciex, Framinghan, MA, USA), SDS-PAGE (BioRad), spectrophotometer (Jasco V-630)*
Data format	*Analyzed*
Experimental factors	The recombinant pea mitochondrial PrxII F (PsPrxII F) was incubated with S-nitrosylating agents 5 mM GSNO (S-nitrosoglutathione) and the NO donor 250 μM SNP (sodium nitroprusside dehydrate) for 30 min at 30 °C.
Experimental features	The thermal aggregation of citrate synthase was evaluated by light scattering at 650 nm during 1800 s at 45 °C.
	*S*-nitrosylated PrxII F was immunoprecipited using anti-biotin antibody and then was subjected to SDS-PAGE. The visualized bands were digested and the peptides were analysed by nano LC ESI–QTOF tandem MS and MALDI peptide mass fingerprinting analysis
Data source location	All the analysis were performed in Spain. MS analysis was carried out in the National Biotechnology Center (CSIC, Spain).
Data accessibility	The data are provided in this article.

## Value of the data

• The data presented here are an advance in the knowledge of the identification of Cys residues targets of *S*-nitrosylation in mitochondrial PrxII F.
• A novel function for *S*-nitrosylated PrxII F inhibiting the citrate synthase thermal aggregation is demonstrated, which could be biologically important under stress situations.

## 1 Data, experimental design, materials and methods

**Fig. 1:**
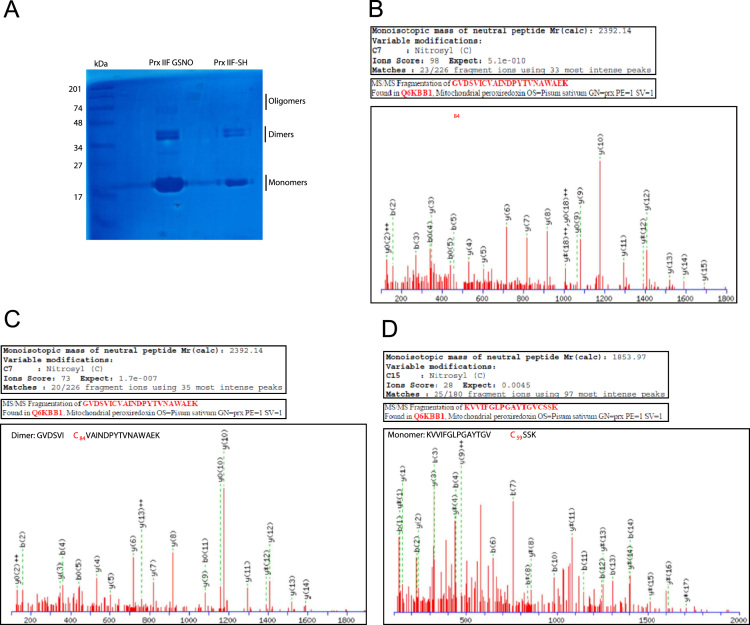
Electrophoretic mobility on SDS-PAGE visualized by silver staining of the recombinant PsPrxII F treated with 5 mM GSNO (PrxII F GSNO) and 50 mM DTT (PrxII F-SH) (A). Peptide analysis were carried out by nano LC ESI–QTOF tandem MS analysis using an Eksigent 1D-nanoHPLC coupled to a 5600 Triple TOF QTOF mass spectrometer (ABSciex, Framinghan, MA, USA). Peptides identified as susceptible of modification (nitrosyl group) in the monomeric form in the Cys 84, GVDSVIC84VAINDPYTVNAWAEK (B); in the monomeric form on the Cys 59 KVVIFGLPGAYTGVC59SSK (C), and dimeric form on the Cys 84 GVDSVIC84VAINDPYTVNAWAEK (D).

**Fig. 2:**
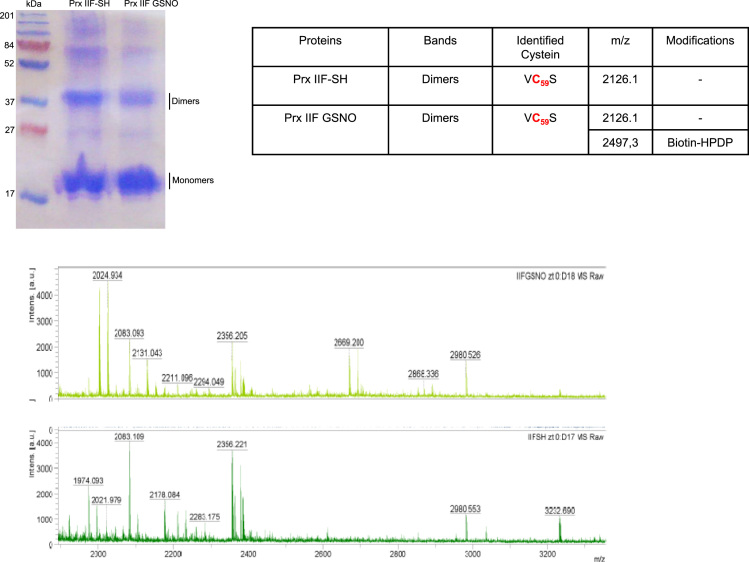
Electrophoretic mobility on SDS-PAGE visualized by silver staining of the recombinant PsPrxII F treated with 5 mM GSNO (PrxII F GSNO) and 50 mM DTT (PrxII F-SH). The table shows the *m*/*z* of the Cys 59 identified as target of modification by HPDP-biotin. MS and MS/MS spectra were obtained by UltrafleXtreme MALDI-TOF/TOF mass spectrometer (Bruker-Daltonics) in auto-mode using Flex Control v3.4 (Bruker-Daltonics).

PsPrxII F treated with 5 mM GSNO was run in SDS-PAGE and the visualized bands were analysed by TOF QTOF mass spectrometry (Fig. 1). *S*-nitrosylated PrxII F was subjected to the biotin switch method [2] and immunoprecipited using anti-biotin antibody and then was subjected to SDS-PAGE. The visualized bands were excised from the gel and digested overnight, in darkness at 37 °C, adding recombinant trypsin (Sigma-Aldrich, St-Louis, MO) at a 1:20 ratio as described in [3]. The digestion was stopped by adding 0.5% trifluoroacetic acid (TFA, Pierce Rockford, IL) and the peptides were extracted immediately, dried by speed-vacuum centrifugation and resuspended in 5 µL of initial ESI solvent solution (100% water+0.5% formic acid). The visualized bands were digested and the peptides were analysed by nano LC ESI–QTOF tandem MS and MALDI peptide mass fingerprinting analysis. The *S*-nitrosylation was estimated by changes in the mass of the peptide containing the Cys residues, GVDSVI**C**_**84**_VAINDPYTVNAWAEK in the monomeric (score 98) and dimeric (73) forms of the PrxII F while KVVIFGLPGAYTGV**C**_**59**_SSK only in the monomeric form (28). Additionally, the nitrosylation of PrxII F was also verified as the incorporation of a HPDP-biotine on the Cys 59 in the dimeric form (Fig. 2).

**Fig. 3:**
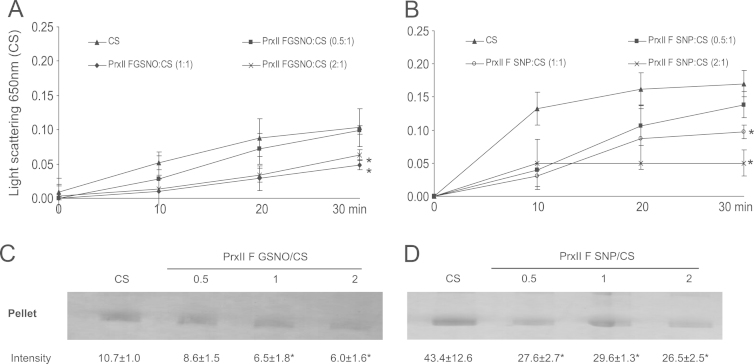
Chaperone activity estimated from the ability of PsPrxII F to inhibit the thermal aggregation of citrate synthase (CS). PrxII F treated with 5 mM GSNO (A) and 250 µM SNP (B) was incubated with CS at a molar ratio (0.5:1, 1:1, 2:1). Samples were separated into pellet and soluble fractions and equal volumes were analysed by SDS-PAGE and visualized by silver staining. Thermal aggregation is evident from the amount of CS present in the pellet fraction after of incubated with GSNO (C) and SNP (D) at the molar ratios (0.5:1, 1:1, 2:1). The numbers represent the intensity of the bands quantified on an image analyser (Gen Tools, Syngene Frederick, MD). The significance of differences between means values was determined by one-way analysis of variance. Duncan׳s multiple range test was used to compare the means when necessary. All error bars represent standard error (SE) of the mean. The asterisk above the bars indicates significant difference (*P*<0.01).

**Fig. 4:**
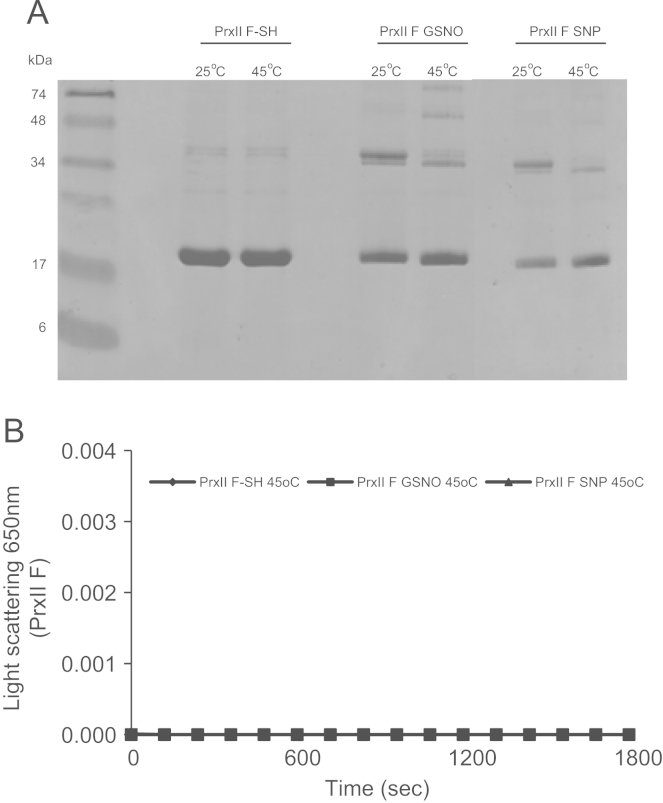
Electrophoretic mobility on SDS-PAGE visualized by Coomasie staining of the recombinant PsPrxII F treated with 50 mM DTT (PrxII F-SH), 5 mM GSNO (PrxII F GSNO) and 250 μM SNP (PrxII F SNP) incubated at 25 °C and 45 °C during 30 min (A). Light scattering at 650 nm of PrxII F treated with DTT, GSNO and SNP incubated at 45 °C during 1800 s (B).

The thermal aggregation of CS was evaluated by light scattering at 650 nm during 1800 s at 45 °C, in the presence of GSNO (Fig. 3A) and SNP-treated (Fig. 3B) PsPrxII F. This was assayed at molar ratio 0.5:1 and 2:1 (PsPrx IIF:CS) observing that it was also prevented at molar ratio 2:1 (PsPrx IIF:CS) while no inhibition of CS aggregation was observed at molar ratio 0.5:1 (PsPrx IIF:CS) and DTT treated-PsPrxII F. Additionally, the samples were separated into a soluble and pellet fraction and visualized on SDS-PAGE gel to identify the CS amount precipitated as consequence of its thermal aggregation. Staining of bands corroborated the results observed at 650 nm, a decrease in the intensity of the bands corresponding to pellet fraction indicated that CS thermal aggregation was prevented by GSNO and SNP-treated PsPrxII F at molar ratios 1:1, 1:2 (PsPrxII F:CS) (Fig. 3C and D). It is noted, that the thermal aggregation of CS assayed on SDS-gel was strongly prevented at molar ratio 2:1 (PsPrxII F:CS) in presence of GSNO-treated PsPrxII F, while SNP-treated PsPrxII F prevented the thermal aggregation of CS to a slow molar ratio of 0.5:1 (PsPrxII F:CS). Experimental controls were carried out to demonstrate that PsPrxII F treated with DTT did not prevent the CS thermal aggregation and to verify that high temperature (45 °C) did not provoke the aggregation of the PsPrxII F (Fig. 4A and B).

## Conflicts of interest

None.
